# Molecular identification of haemoparasites in animals using blood lysate PCR: a quick and inexpensive alternative to purified whole genomic DNA

**DOI:** 10.1080/10495398.2024.2390935

**Published:** 2024-08-13

**Authors:** Binod Kumar, Nilima N. Brahmbhatt, Bhupendrakumar Thakre, Biswa Ranjan Maharana, Vijay L. Parmar, Manoj Kumar

**Affiliations:** a Department of Veterinary Parasitology, College of Veterinary Science and Animal Husbandry, Kamdhenu University, Junagadh, India; b Referral Veterinary Diagnostic and Extension Centre, LUVAS, Karnal, India; c Department of Veterinary Medicine, College of Veterinary Science and Animal Husbandry, Kamdhenu University, Junagadh, India; d Central Animal House, Indira Gandhi Institute of Medical Sciences, Patna, India

**Keywords:** Blood lysate PCR, haemoparasites, betaine, molecular diagnosis, cost analysis

## Abstract

Haemoparasitic diseases constitute a significant constraint to economic livestock farming. Diagnostic techniques that are inexpensive, rapid, reliable, and precise are crucial for the management of diseases. In this context, PCR assays are very valuable yet expensive since the samples must be processed before being included in the PCR reaction. Accordingly, the goal of the current study was to lower the PCR costs without jeopardizing the assay’s sensitivity and specificity. For that purpose, the alkaline solution was optimized for low cost and quick DNA extraction (blood lysate), and PCR reagents were modified for optimum reaction. In comparison to purified whole blood genomic DNA, the currently developed and optimized blood lysate method was found to be 95.5% less expensive, as well as being equally sensitive and specific for the molecular detection (PCR) of haemoparasites like *Babesia, Theileria*, *Trypanosoma* and rickettsiales in cattle, buffaloes, horses, and dogs. The assay was also demonstrated to be quick, less likely to cross-contaminate, and appropriate for use in laboratories with limited resources. Therefore, the currently developed and optimized blood lysate method could serve as a viable alternative to purified whole blood genomic DNA for molecular detection (PCR) of haemoparasites in animals particularly in resource-limited settings.

## Introduction

Haemoparasites such as *Babesia, Theileria, Trypanosoma, Anaplasma,* and *Ehrlichia* cause diseases in animals and are considered serious constraints to the health and productivity of domestic animals in tropical and subtropical regions of the world. Some hemoparasites are important zoonotic agents that infect and cause mild to severe illnesses in humans. The effective control and treatment of diseases require rapid, reliable, and sensitive diagnostic tests, which can also serve to monitor the effectiveness of the therapeutic and prophylactic protocol. Due to their simplicity and low cost, traditional diagnostic methods like microscopy are frequently used in parasite diseases. However, they have significant drawbacks, particularly when it comes to the detection of haemoparasites, such as low sensitivity and specificity, the need for skilled personnel, and time-consuming procedures.^1–4^ Similarly, the use of sero-diagnostics is constrained by poor antigen quality and cross-reactivity.^2^

With the emergence of DNA-based technology, it has now become easier to detect, and characterize haemoparasites, and a significant number of samples can be processed quickly. Additionally, it can overcome the major drawbacks of microscopy, clinical diagnosis and serodiagnostics.^2^^,^^4^^,^^5^ However, a significant constraint remains the operating cost associated with this method.The conventional PCR (cPCR) assay is completed in three steps: 1. Isolation of DNA, 2. PCR reaction and 3. visualization of DNA through agarose gel electrophoresis. Of the three steps involved, the isolation of DNA incurs the highest cost, and it is pivotal as it significantly influences the outcome of the process.Directly using body fluids in PCR poses challenges due to the presence of several PCR-inhibitory components, particularly in blood. These components include proteins, polysaccharides, lipids, and their conjugates, which can interfere with the PCR process.^6^^,^^7^ Hence, before PCR assay, DNA extraction from biological samples is essential to mitigate the inhibitory effects of various components present, ensuring accurate and reliable results. The Phenol-Chloroform method^8^ is the most widely used and basic technique for isolating DNA from biological materials. It entails several steps, including pre-treating the sample with protease/detergent, extracting the DNA with phenol/chloroform, precipitating the DNA with ethanol, and re-dissolving the DNA in the buffer. There is a multitude of commercially available DNA separation kits for isolating purified DNA that are based on silica filters, silica particles, magnetic particles, or protein-aggregating agents. Although they simplify DNA isolation compared to prior methods, they are still expensive and time-consuming. Additionally, the isolation process typically increases the risk of sample contamination with foreign DNA, potentially including genetic material from previous samples.^6^

Initially, efforts were made to address these challenges by attempting to use blood directly in PCR following minimal processing, such as washing blood cells in buffer, diluting blood in sterile water, or treating with an alkaline solution to amplify the animal/host-associated DNA fragments.^6^^,^^7^^,^^9–12^ Subsequently, modifications were made to utilize dried blood spots on various substrates like FTA cards, filter paper, or Micro cards for DNA amplification of either the host or the blood parasites. However, despite these modifications, this method remains somewhat time-consuming, expensive, and involves multiple steps compared to the streamlined approach described here.^7^^,^^13–15^ Indeed, this approach is increasingly common in forensic science as well, to extract information from minute quantities of biological materials.^16^ The recently developed direct blood PCR assay amplifies either the host DNA or the DNA of the parasites by using a few microliters of blood directly in specific PCR reagents/PCR master mix.^17–20^ These methods are reported to be precise and sensitive but are still expensive. Therefore, looking at the significance and growing use of molecular techniques in the detection of foreign bodies (pathogens, etc.), research and development should be taken to further reduce the cost and simplify the process. Accordingly, the goal of the current research is to develop, standardize, and evaluate quick and low-cost novel blood processing methods for the molecular detection of common haemoparasites in animals.

## Materials and methods

### Samples

The blood samples were collected from sick cattle, buffaloes, horses, and dogs presented at various animal hospitals in and around Junagadh city for treatment during the years 2018–2022. Further, the samples were submitted to the Department of Veterinary Parasitology for the screening of the haemoparasitic infections, if any. The whole blood was used for the isolation of genomic DNA and optimization and validation of blood lysate PCR. The data related to individual samples including place, species, breed, and date of collection were recorded in a proforma. Blood samples collected from a healthy calf maintained at a cattle breeding farm (CBF), Junagadh Agricultural University, Junagadh was used as negative control while microscopically positive blood for *Babesia, Theileria*, *Trypanosoma evansi, Anaplasma marginale,* and *Ehrlichia canis* was used as positive control.

### Extraction of genomic DNA

One hundred fifty micro-liter of genomic DNA was extracted from a 200 µl whole blood sample using a whole blood genomic DNA purification kit (Qiagen, Hilden, Germany) as per the manufacturer’s protocol. The concentration of the whole blood genomic DNA was measured spectrophotometrically, and it was then stored at −20 °C until usage.

### Amplification of the GAPDH (glyceraldehyde-3-Phosphate dehydrogenase) gene in domestic animals

Oligo-primers were designed from the conserved region of the nucleotide sequences accessible at GenBank (NCBI, USA) (XM_014482068; XM_010844969; BC102589; XM_060411594; XM_006065800; HG994413; AJ431207; NM_001163856) using bioinformatics tools (GeneTools, USA and Primer-BLAST, NCBI, USA), and custom synthesized from a commercial service provider (Eurofins Genomics India Pvt. Ltd., Bangaluru, India) to amplify a portion of the GAPDH gene of the host (cattle, buffaloes, horses, and dogs). The oligos are GAPDH-F: 5′gcgccaagagggtcatcatc3′ (nt 189–208) and GAPDH-R: 5′ggggccatccacagtcttct3′ (nt 670–689) as per GenBank Accession AJ786261. A 25 µl PCR reaction was set up using 12.5 µl of DreamTaq Green PCR master mix (Thermo Scientific, Vilnius, Lithuania), 1 µl each of forward and reverse PCR primers (10 mM), 2 µl template DNA (purified DNA) and remaining nuclease-free water (NFW). A reaction mixture without template DNA was used as a negative control. The reaction mixture was incubated in a Gradient thermal cycler (Applied BioSystem, USA) at the optimized PCR condition: initial denaturation at 95 °C for 3 min followed by 32 cycles of denaturation (95 °C for 20 s), annealing (60 °C for 30 s) and extension (72 °C for 30 s). The final extension was kept at 72 °C for 5 min. Subsequently, 10 µl of PCR product along with DNA ladder (Thermo Scientific, Vilnius, Lithuania) was loaded in 1.2% agarose gel containing ethidium bromide (0.5 µg/ml) and electrophoresis was done at 100 V for 40 min. The DNA on the gel was visualized and documented using Gel Doc (Syngene, USA).

### Amplification of targeted DNA fragments of various haemoparasites of animals

The laboratory-standardized PCR primers (Table 1) were used in the present study for the identification of haemoparasitic infections in animals through PCR assay. The standard 25 µl PCR reaction was set up in 200 µl PCR tubes as mentioned in the previous section. The haemoparasite positive DNA was used as positive control while without template DNA was used as negative control. The reaction mixture was incubated in a Gradient thermal cycler (Applied Bio System, USA) at standard PCR conditions as Initial denaturation (95 °C for 5 min), 35 cycles of denaturation (96 °C for 15 s), annealing (as per the primers, see Table 1) and extension (as per the primers, see Table 1) and final extension (72 °C for 5 min). After the reaction, 10 µl of PCR products were loaded in 1.2% agarose gel containing ethidium bromide (0.5 µg/ml) and electrophoresis was done at 100 V for 40 min. The DNA on the gel was visualized and documented using Gel Doc (Syngene, USA).

**Table 1.** Details of PCR primers used for identification of various haemoprotozoans of animals through PCR assay.

Primer ID	Sequence (5’-3’)	Identify	Targeted DNA fragmentes	Amplicon size	Annealing temperature (AT) and Extension time	Reference
BaF (Forward)	aatacccaatcctgacacaggg	Piroplasm (*Babesia/ Theileria*)	18S rRNA	about 400 bp	AT (58 °C for 30 s); Extension (72 °C for 40 s)	4
BaR (Reverse)	ttaaatacgaatgcccccaac					
MRCF (Forward)	cgcttgcctcattatcgcac	*B. bigemina*	Rap-1c	462 bp		4
MRCR (Reverse)	cctcccctcttgaaacgcat					
Tctb1 (Forward)	actttggccgtaatgttaaac	*T. annulata*	Cytochrome b	312 bp		21
Tctb2 (Reverse)	ctctggaccaactgtttgg					
Tom5 (Forward)	ctttgcctaggatacttcct	*T. orientalis*	MPSP	776 bp		22
Tom6 (Reverse)	acggcaagtggtgagaact					
Tf3 (Forward)	gcacaaatgccgacggta	*Trypanosoma evansi*	RoTat1.2	200 bp		23
Tb3 (Reverse)	gtcgttgccggttattgct					
ECAN5 (Forward)	caattatttatagcctctggctctggctatagga	*Ehrlichia canis*	16S rRNA	389 bp	AT (52 °C for 30 s); Extension (72 °C for 30 s)	24
HE3 (Reverse)	tataggtaccgtcattatcttccctat					
Ana290F (Forward)	atgccgggcactttaaggaa	(Rickettsials)*Anaplasma*	16S rRNA	290 bp	AT (58 °C for 30 s); Extension (72 °C for 30 s)	Present study
Ana290R (Reverse)	taagccaattcccatggcgt					

### Optimization of blood lysate PCR on host GAPDH gene

Initially, the GAPDH gene of the host was amplified to acquire baseline data on the effective concentration of NaOH to be utilized in the preparation of blood lysate (mainly dogs). Accordingly, 10 µl of apparently healthy animal blood was arbitrarily included in 0.6 ml tubes containing 200 µl of NaOH solution of varying concentrations starting with 5 mM to 80 mM with a difference of 5 mM. One test tube containing 200 µl of nuclease-free water was also kept as a negative control. The mixture was vortexed for a few seconds and incubated in a water bath maintained at 90 °C ± 5 °C for 10 min. After incubation, the mixture was vortexed for a brief period and centrifuged at 6000×*g* for 5 min. Now, this blood lysate (4 µl) was used as the template in place of the purified DNA in a 25 µl PCR reaction as mentioned in the previous section. The blank 25 mM NaOH was included in PCR as a negative control. The PCR reaction was performed at optimized thermocyclic conditions and 10 µl of PCR product along with DNA ladder (Thermo Scientific, Vilnius, Lithuania) was electrophoresed, visualized and documented in Gel Doc (Syngene, USA).

### Optimization of blood lysate PCR on piroplasm 18S rRNA gene

**Figure 1.**
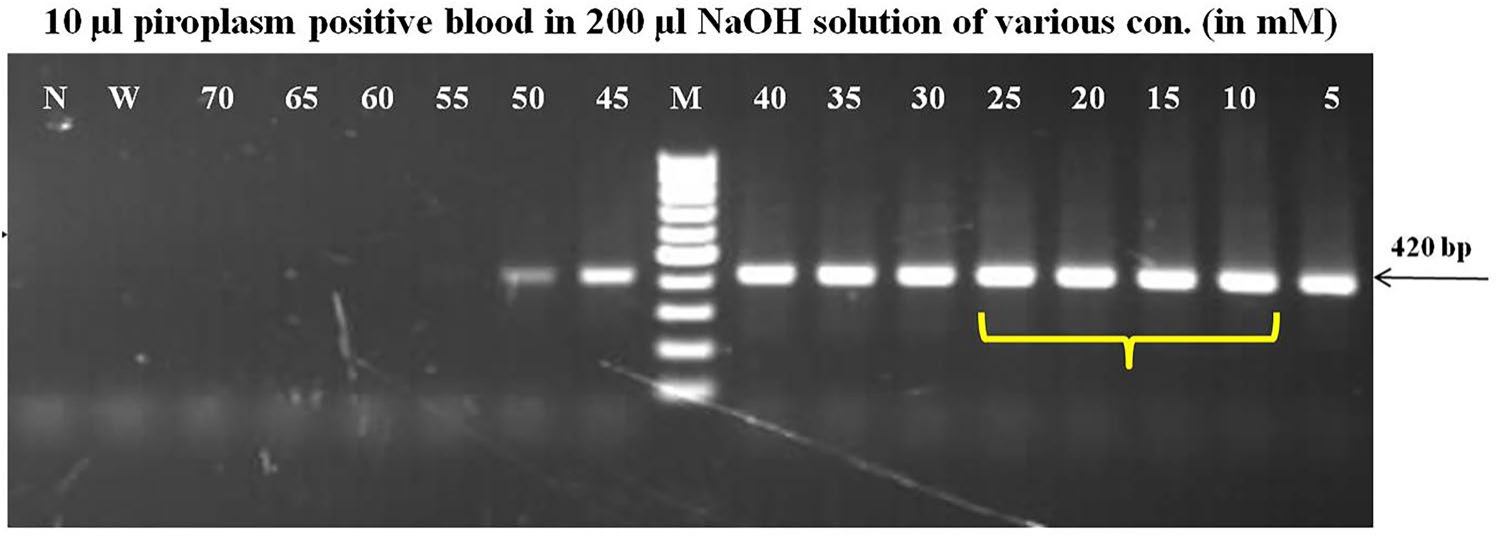
Optimization of concentration of NaOH for preparation of blood lysate to amplify the targeted gene of piroplasm (18S rRNA) under standard PCR condition (lanes: 5–70 mM NaOH concentrations, M – 100 bp DNA ladder, W – water, N – negative control).

The microscopically *Theileria* positive blood sample was used to standardize and optimize the methodology. The concentration of NaOH and the amount of blood used for the preparation of blood lysate, as well as the quantities to be included in a PCR alongside Betaine (as a PCR enhancer), primers, and MgCl2 concentration, were optimized by amplifying the 18S rRNA gene of microscopically positive *Theileria* blood samples. The PCR primers BaF/BaR (Table 1) were used to amplify about 410 bp of 18S rRNA gene fragments of piroplasm.^4^ Firstly, according to the results obtained from host (dog) GAPDH gene fragment amplification, the blood lysate was prepared by including 10 µl *Theileria* positive blood samples in 200 µl of 5–70 mM NaOH (Fig. 1). The 200 µl NFW was used instead of NaOH solution as NaOH control. The obtained lysate (4 µl) was used in a 25 µl PCR reaction containing 12.5 µl of 2x DreamTaq Green PCR Master Mix (Thermo Scientific, Vilnius, Lithuania), 1 µl each of 10 mM forward and reverse primers (BaF/BaR) and 6.5 µl of NFW to amplify the targeted DNA fragment of piroplasm. The PCR reaction was performed in a Thermal cycler (Applied Bio System, USA) for 35 cycles of denaturation (96 °C for 15 s), annealing (58 °C for 30 s), and extension (72 °C for 30 s), and 5 min each of initial denaturation at 95 °C as a first step and final extension at 72 °C at the end. Secondly, to optimize the concentrations of primers, MgCl_2_, Betaine (Sigma, USA) and the amount of blood lysate to be used, the variable concentrations of each were included in 25 µl PCR reactions such as each primer: 10 pmol, 15 pmol, 20 pmol and 30 pmol concentration; additional MgCl_2_ (2X DreamTaq Green PCR Master Mix contain 4 mM MgCl_2_): 0 mM (not added), 1.5 mM and 2 mM; Betaine: 0.4 M, 0.6 M, and 0.8 M; and blood lysate as template DNA: 4 µl, 5 µl and 6.5 µl obtained from 10 µl of *Theileria* positive blood in 200 µl of 25 mM NaOH (maximum concentration NaOH where best amplification of 18S rRNA was obtained) (Fig. 1). The PCR products were loaded (10 µl each) in 1.2% agarose gel, electrophoresed and amplification was documented. The amplicons were analyzed of and the best PCR reaction condition was identified. Subsequently, the optimal PCR reaction mixture was used to optimize the concentration of NaOH and the amount of blood to be included for the preparation of blood lysate. Various concentrations of NaOH (10, 15, 20, and 25 mM) and *Theileria* positive blood (10, 20, 30 and 40 µl) were used to prepare the blood lysate. The obtained lysate was included as a template in a standardized PCR reaction as mentioned above. The 10 µl of each PCR product was loaded in 1.2% agarose gel, electrophoresed and documented using Gel Doc (Syngene, USA) as mentioned in the previous section. Furthermore, PCR reactions were carried out at weekly intervals for up to a month to observe the self-life/reactive potentiality of blood lysate maintained at room temperature. The reactivity of haemoparasite positive blood stored at 4 °C (up to 15 days), − 20 °C and −80 °C was also assessed at optimized conditions.

### Sensitivity of the blood lysate PCR in comparison to PCR on purified whole blood genomic DNA isolated by commercial blood DNA isolation Kit

Microscopically positive *T. annulata*, *T. orientalis, B. bigemina,* and *T. evansi* blood samples were serially diluted with blood samples collected from the healthy cow (haemoprotozoan negative in both microscopy and PCR test) maintained at Cattle Breeding Farm (CBF) of the institute following the method as described by Wang *et al.*^25^ to access the sensitivity of the PCR assays. From the blood mixture, blood lysate was prepared and whole blood genomic DNA was isolated. The 18S rRNA gene of piroplasm (using BaF/BaR primers), cytochrome b gene of *T. annulata* (using Tctb1/Tctb2 primers, Table 1) and MPSP gene of *T. orientalis* (using Tom5/Tom6, Table 1) were amplified from ten-fold serially diluted *Theileria* infected blood and to amplify the rap1-c gene of *B. bigemina* (using MRCF/MRCR primers, Table 1) and RoTat1.2 gene of *T. evansi* (using Tf3/Tb3 primers, Table 1) five-fold serially diluted *B. bigemina* and *T. evansi* infected blood were used having the parasitaemia of about 1.0 to 2.0%. Total genomic DNA was extracted from each diluted blood sample using a commercial Blood DNA extraction Kit (QIAamp DNA Mini Kit, Qiagen, Hilden, Germany) in 150 µl elution buffer. The optimized PCR reactions for blood lysate (12.5 µl of 2x PCR Master Mix, 2 µl each of 10 mM forward and reverse primers, 2 µl 5 M Betaine, 4 µl blood lysate, and 2.5 µl of NFW) and purified DNA (12.5 µl of 2x PCR Master Mix, 1 µl each of 10 mM forward and reverse primers, 4 µl template DNA and 6.5 µl of NFW) were used to amplify the targeted genes. The amplifications were compared by loading 10 µl of PCR product in 1.2% agarose gel, electrophoresed and documented using Gel Doc (Syngene, USA) as described previously.

### Comparison of PCR assay on field samples

Blood samples (*n* = 1387) of domestic animals such as cattle, buffaloes, horses, and dogs received for diagnosis at the Department of Veterinary Parasitology and VCC laboratory during 2018–2022 were randomly included in the present study. The total genomic DNA was extracted using commercial Kit (DNASure Blood Mini Kit, Nucleo-pore, Genetix, New Delhi, India; QIAamp DNA mini kit, Qiagen, Hilden, Germany; HiPurA™ Blood Genomic DNA Miniprep Purification Kit, Himedia, Thane, India; GeneJET Whole Blood Genomic DNA Purification Mini Kit, Thermo Scientific, Vilnius, Lithuania) in 150 µl of elution buffer and stored at −20 °C till further use. The quality of isolated whole blood genomic DNA was assessed through 1% agarose gel electrophoresis. For blood lysate PCR, blood samples were either immediately processed or kept in the refrigerator at 4 °C till further use. Both, the PCR assays were conducted concurrently on the same samples at optimized conditions. Moreover, microscopically positive and negative samples were included in the present experiment (microscopically 482 positive and 905 negative for haemoparasitic infections).

Moreover, to confirm the specific reactivity of the PCR primers with blood lysate and purified blood DNA, randomly, amplified PCR products of a few samples along with primers were submitted to the commercial service provider (Eurofins Genomics India Pvt. Ltd., Bengaluru, India) for bi-directional Sanger sequencing. Upon receiving of sequences, the quality of sequences was checked in BioEdit programme, both forward and reverse sequences were aligned, ends of sequences were trimmed to remove the poor quality or miss-match nucleotides, and a correct consensus sequence was obtained. Subsequently, the sequence analysis and similarity searches were performed with the BLASTn (NCBI, USA), and species were confirmed. The sequences generated from different species of parasites and animals were submitted to GenBank (NCBI, USA), and accession numbers were obtained.

### Comparison of blood processing costs

To compare the cost of two blood processing methods and PCR assays, the value of the standard kits, reagents, and other consumables were considered. The average costs of DNA isolation kits along with required plastic ware, chemicals, instrumentation, etc. were compared with the cost of materials required for blood lysate PCR (Table 2).

**Table 2.** Comparative analysis of cost of PCR on blood lysate and purified DNA.

DNA extraction by kit			Blood lysate method		
Manufacturer	Cost of kit^a^ (250 reactions)	Cost per sample (in Indian rupees)	Reagents (mol. bio. grade)	Unit cost^a^	Cost per sample (in Indian rupees)
Genetix, India	32083.00	128.33	10 M NaOH, Himedia, India	690.00 per 100 ML	0.02
Thermo Scientific, Lithuania	51505.00	206.00			
Himedia, India	31875.00	127.50	Nuclease free water (Himedia, India)	2009 per 1000 ML	0.40
Qiagen, Germany	32980.00	131.92			
Kit Cost per sample (Av.)		148.44	Cost per sample		0.42
Plastic wares^b^ and other consumables					
Tips (*n* = 7)		21.00	Tips (*n* = 2)		6.0
1.5 ml tubes (*n* = 2)		4.00	0.6 ml tubes (*n* = 1)		1.5
Absolute Ethanol (200 µl)		2.0	–		–
Final cost of DNA extraction per sample		175.44	Final cost of blood lysate preparation per sample		7.92
Cost of PCR reaction (PCR master mix, primers, plastic wares, etc.)		53.50	Cost of PCR reaction (PCR master mix, primers, plastic wares, Betaine, extra primers, etc.)		61.50
Final PCR Cost /sample /reaction		228.94	Final PCR Cost /sample /reaction		69.42

^a^Prices are in Indian rupees as per the year 2019–2020 (excluding taxes). ^b^The average price of plastic wares was included from standard manufacturer’s (Tarson, India; Genaxy, India).

### Statistical analysis

The PCR tests used to detect haemoparasites in animal blood samples were compared using the Chi-square (χ^2^) test and a difference of *p* ≤ 0.05 was deemed significant.

## Results

### Amplification of GAPDH gene of domestic animals

The GAPDH gene fragments were amplified successfully using whole-blood genomic DNA that was taken from cattle, buffaloes, horses, and dogs. In contrast to the two bands of amplicons (501 and 230 bp) evident on the gel for cattle, buffaloes, and horses, just one band (230 bp) of extremely specific amplification was reported for dog blood samples (Supplementary Fig. 1).

### Amplification of targeted haemoparasites DNA found in the blood of animals

Purified whole blood genomic DNA isolated from microscopically positive samples was amplified without eliciting any nonspecific responses. This amplification yielded targeted DNA fragments ranging from 200 to 776 base pairs in size, representing various haemoparasites (Supplementary Fig. 2).

### Optimization of blood lysate PCR

**Figure 2.**
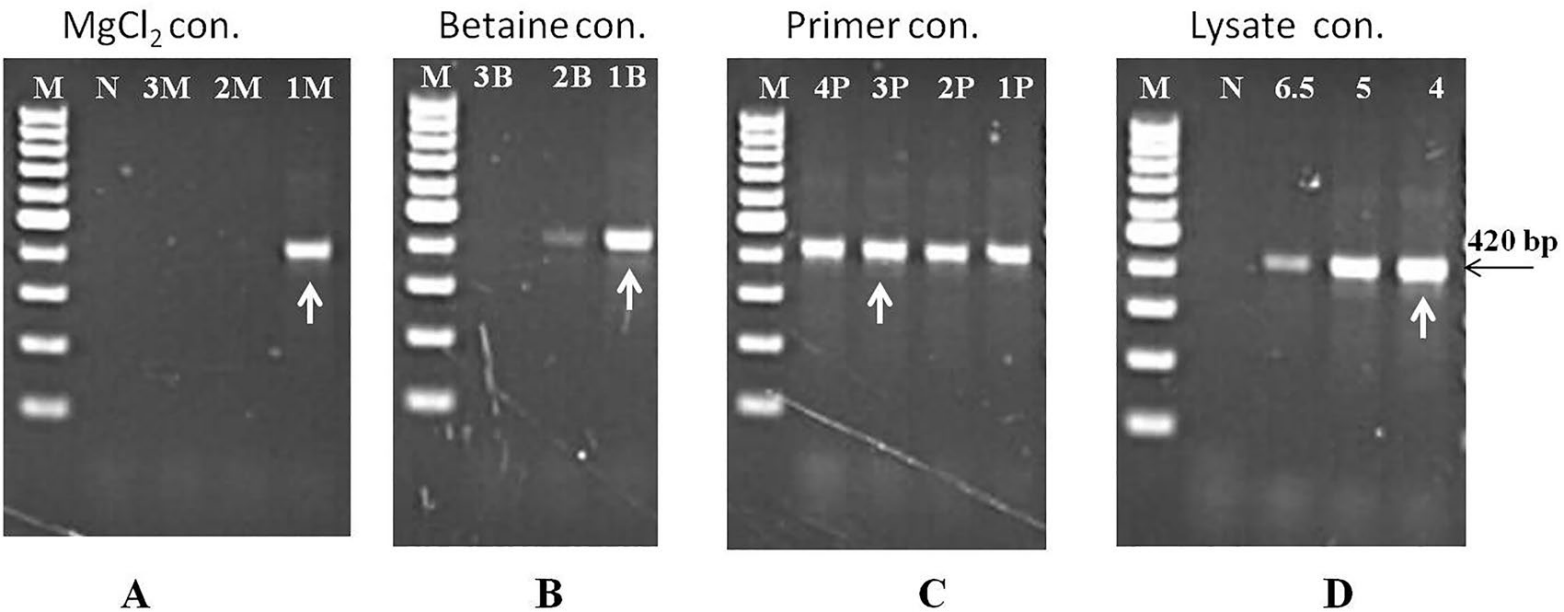
Optimization of PCR for amplification of 18S rRNA gene of piroplasm (*Theileria*) using blood lysate. (A) Optimization of MgCl_2_ (1 M – no additional MgCl_2_, 2 M – 1.5 mM, 3 M – 2 mM, N – NTC and M – 100 bp DNA ladder), (B). Optimization of betaine (1B – 0.4 M, 2B – 0.6 M, 3B – 0.8 M and M – 100 bp DNA ladder), (C). Optimization of primer concentration (1 P – 10 pmol, 2 P – 15 pmol, 3 P – 20 pmol, 4 P – 30 pmol each and M – 100 bp DNA ladder) and (D). Optimization of blood lysate in 25 µl reaction (4, 5, and 6.5 µl, N – NTC and M – 100 bp DNA ladder).

**Figure 3.**
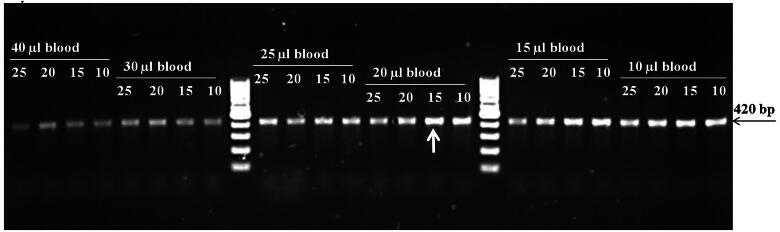
Optimization of blood quantity (10, 15, 20, 25, 30 and 40 µl) at four different concentration of NaOH (10, 15, 20, 25 mM) based upon the amplification of 18S rRNA gene of piroplasm for the preparation of blood lysate. (100 bp DNA ladder).

The amplification of the 230 bp fragment of the dog’s GAPDH gene was recorded at all studied concentrations (5–80 mM) of NaOH and even in nuclease-free water. The amplification was progressively increased up to 25 mM NaOH concentration, maintained for the next 3–4 concentrations, exhibited maximum at 55 mM NaOH and then subsequently decreased to very faint amplification in 80 mM NaOH (Supplementary Fig. 3). Similarly, specific amplification of 18S rRNA gene fragment of *Theileria*/piroplasm was recorded on blood lysate prepared from microscopically positive *Theileria* blood samples up to 50 mM NaOH concentration. However, comparatively better amplification was observed from 10 to 25 mM NaOH. No amplification was observed in blood lysate prepared in NFW (Fig 1). By optimizing the PCR reagents, the 18S rRNA gene (BaF/BaR primer) of piroplasm was amplified to its highest quality possible. This has been achieved by employing a PCR reaction mixture containing 12.5 µl of 2x DreamTaq Green PCR Master Mix (Thermo Scientific, Lithuania), 2 µl each of 10 mM forward and reverse primers, 2 µl of 5 M Betaine, 4 µl blood lysate and 2.5 µl of NFW devoid of any nonspecific reaction (Fig. 2). Consequently, the best amplification of the targeted gene of piroplasm was obtained in blood lysate prepared by dissolving 20 µl of positive blood in 200 µl of 15 mM NaOH and heating at 90 °C ± 5 °C for 10 min (Fig. 3). The positive reactivity of blood lysate was found even after one month of storage at room temperature. Furthermore, the targeted amplification of parasitic gene was achieved from microscopically positive blood stored at 4 °C, −20 °C, and −80 °C temperatures. No reaction was observed when blood was lysed in NFW or direct blood was used in the PCR reaction. Amplification of fragments (200–776 bp) of targeted genes was accomplished by using the precise sets of primers that were used to identify the haemoparasites such as *Babesia, Theileria, Trypanosoma, Anaplasma,* and *Ehrlichia* in domesticated animals.

### Sensitivity of blood lysate in comparison to blood DNA isolated by commercial kit

**Figure 4.**
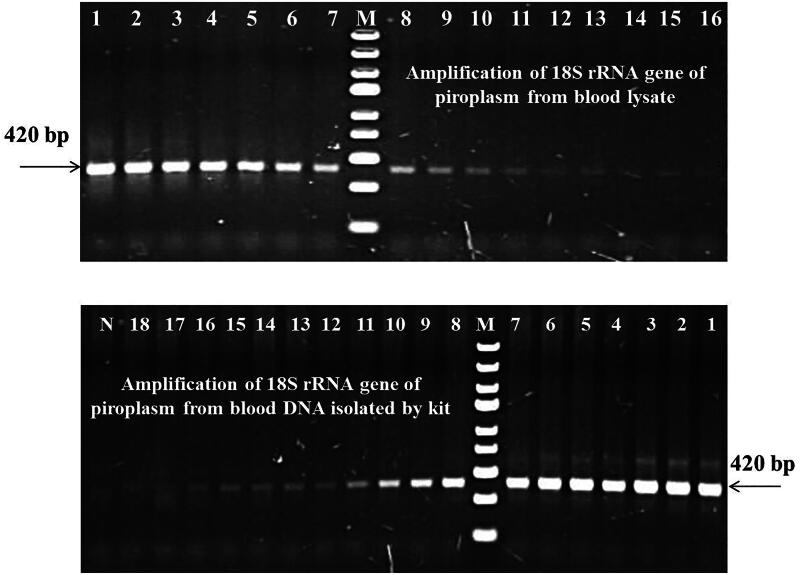
Comparative sensitivity of blood lysate to amplify the 18S rRNA gene of piroplasm of domestic animals. Numbers 1–18: 10-fold serially diluted *T. annulata* positive blood samples, N – NTC, M – DNA ladder (O’GeneRuler express DNA ladder, Thermo Scientific, Lithuania).

The amplification of the piroplasm 18S rRNA gene (using BaF/BaR primers) was recorded up to the 15th and 16th dilution of positive blood using blood lysate and purified DNA, respectively. However, good readability/visibility of bands in both cases was up to the 11th dilution (Fig. 4). Similarly, the amplification of the cytochrome b gene of *T. annulata* and MPSP gene of *T. orientalis* was recorded up to the 10th and 12th dilution in blood lysate; and 11th and 13th dilution in purified DNA, respectively (Supplementary Figs. 4 and 5). Further, amplification of the rap1-c gene of *B. bigemina* and RoTat1.2 gene of *T. evansi* was achieved up to the 7th and 11th dilution in blood lysate; and 8th and 12th dilution in blood DNA isolated by kit, respectively (Supplementary Figs. 6 and 7). In all the cases, the reactivity/sensitivity of blood lysate was reported to be one dilution less than purified blood DNA isolated by a commercial blood DNA isolation kit which is extremely close to being able to detect an extremely low number of parasites in animals’ blood.

### Evaluation of blood lysate PCR in field samples

**Figure 5.**
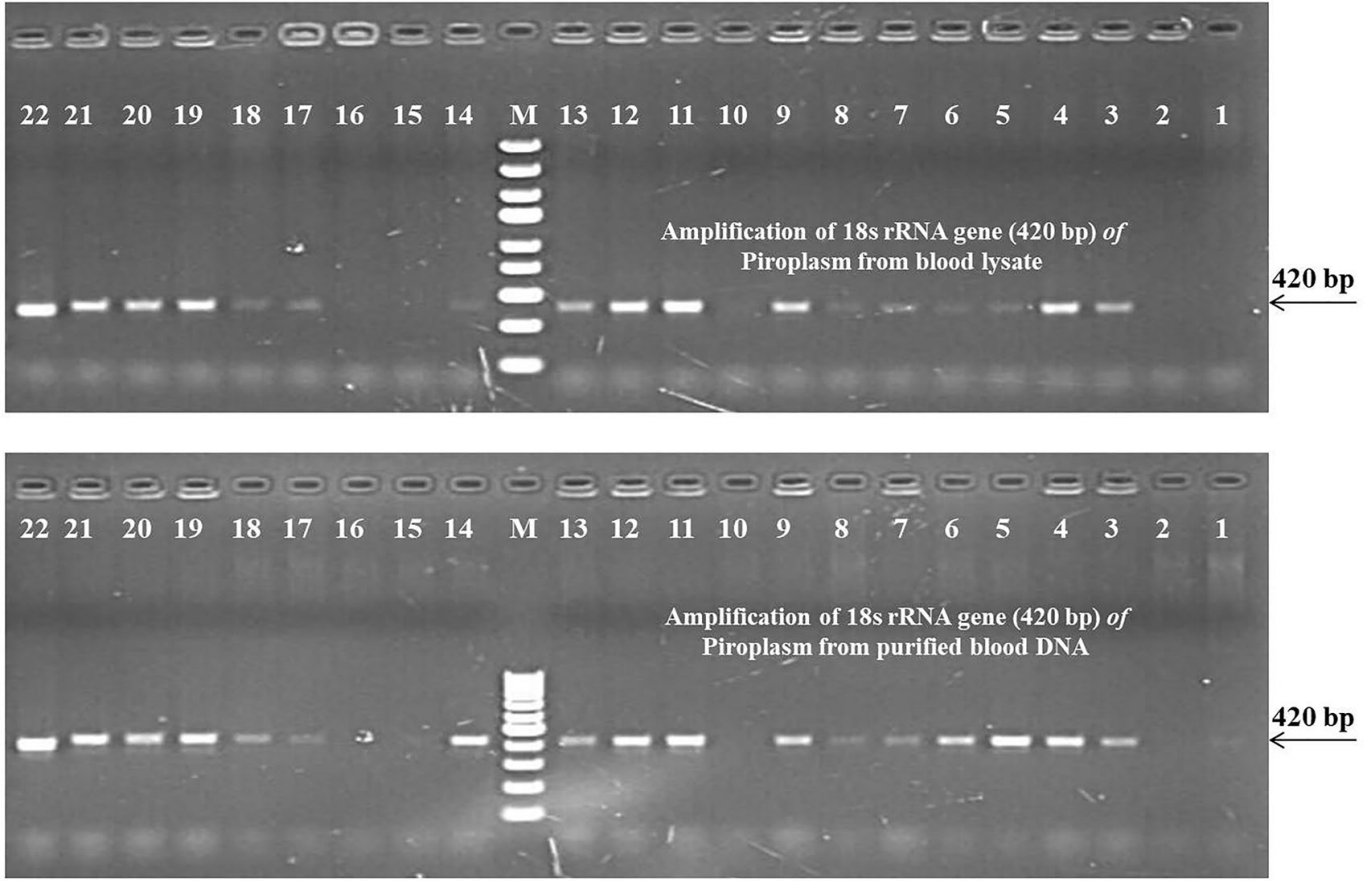
Amplification of 18S rRNA gene of piroplasm from microscopically positive and negative blood samples using both blood lysate and purified whole blood genomic DNA. Samples 11 and 19 to 22 are microscopically positive and all others are microscopically negative. Out of 17 microscopically negative blood samples, 12 samples are showing positivity in PCR by both the methods of DNA isolation. M – DNA ladder (for blood lysate -O’GeneRuler express DNA ladder; and for purified blood DNA – 100 bp DNA ladder, thermo scientific, Lithuania).

All microscopically positive blood samples were equally reacted with both blood lysate and purified DNA to detect various haemoparasites like *T. annulata, T. orientalis, B. bigemina, T, evansi, Anaplasma* and *E. canis*. Therefore, the evaluation of blood lysate PCR was done on microscopically negative blood samples as the sensitivity of the PCR method is higher than microscopy to detect the haemoparasites in the blood (Fig. 5). In microscopically negative blood samples, PCR assay based on purified whole blood genomic DNA detected 29.50%, 22.19%, 19.00%, 3.88%, 6.85%, 7.74%, 14.15% and 13.41% of piroplasm, *T. annulata, T. orientalis, B. bigemina*, *T. evansi*, *Anaplasma,* and *E. canis* infection, respectively whereas blood lysate PCR detected the same pathogens with slightly lower sensitivity for piroplasm (29.06%) and *T. orientalis* (18.72%) but equivocal sensitivity in *T. annulata, B. bigemina*, *T. evansi, Anaplasma,* and *E. canis*. The differences were statistically not significant (*p* > 0.05). The results of PCR on microscopically negative blood samples are presented in Tables 3–6.

**Table 3.** Comparison of purified DNA and blood lysate PCR for detection of piroplasm using BaF/BaR primers in field samples (*n* = 345).

Animals	Microscopically negative samples		
	Total samples	Detection of piroplasm	
		In blood DNA isolated by kit (%)	In blood lysate (%)
Cattle	108	32 (29.63%)	31 (28.70%)
Buffaloes	98	30 (30.61%)	30 (30.61%)
Horses	64	19 (29.69%)	18 (28.13%)
Dogs	75	20 (26.67%)	19 (25.33%)
Total	345	101 (29.28%)	98 (28.41%)

**Table 4.** Comparison of purified DNA and blood lysate in PCR for detection of *T. annulata, T. orientalis* and *B. bigemina* using Tctb1/Tctb2, Tom5/Tom6 and MRCF/MRCR primers, respectively in field samples (*n* = 206).

Animals	Microscopically negative samples						
	Total samples	Detection of *T. annulata*		Detection of *T. orientalis*		Detection of *B. bigemina*	
		In blood DNA isolated by kit (%)	In blood lysate (%)	In blood DNA isolated by kit (%)	In blood lysate (%)	In blood DNA isolated by kit (%)	In blood lysate (%)
Cattle	108	27	27	18	17	5	5
		(25.00%)	(25.00%)	(16.67%)	(15.74%)	(4.63%)	(4.63%)
Buffaloes	98	18	18	22	22	3	3
		(18.37%)	(18.37%)	(22.45%)	(22.45%)	(3.06%)	(3.06%)
Total	206	45	45	40	39	8	8
		(21.84%)	(21.84%)	(19.42%)	(18.93%)	(3.88%)	(3.88%)

**Table 5.** Comparison of use of purified DNA and blood lysate in PCR for detection of *T. evansi* using Tf3/Tb3 primers in field samples (*n* = 155).

	Microscopically negative samples		
		Detection of *T. evansi*	
Animals	Total samples	In blood DNA isolated by kit (%)	In blood lysate (%)
Cattle	49	3 (6.12%)	3 (6.12%)
Buffaloes	32	2 (6.25%)	2 (6.25%)
Horses	40	5 (12.50%)	5 (12.50%)
Dogs	34	2 (5.88%)	2 (5.88%)
Total	155	12 (7.74%)	12 (7.74%)

**Table 6.** Comparison of purified DNA and blood lysate in PCR for detection of *anaplasma* spp. and *Ehrlichia canis* using Ana290F/Ana290R and ECAN5/HE3 primers, respectively in field samples.

Detection of *Anaplasma* in cattle and buffaloes (*n* = 106)		Detection of *E. canis* in dogs (*n* = 75)	
In blood DNA isolated by kit (%)	In blood lysate (%)	In blood DNA isolated by kit (%)	In blood lysate (%)
15	15	10	10
(14.15%)	(14.15%)	(13.33%)	(13.33%)

Sequence analysis revealed that the amplified product obtained from both blood lysate PCR and purified DNA PCR was similar. Therefore, sequences obtained from blood lysate PCR were submitted to GenBank (NCBI, USA) and accessions were generated (Supplementary Table 1).

### Cost analysis

The cost of processing a single blood sample for a PCR test using both methods was calculated, and the blood lysate method was found to be 95.5% less expensive in terms of consumables. However, the cost of a PCR reaction using blood lysate for a sample was about 15% higher than PCR on purified DNA due to additional expenditures in PCR reagents especially betaine in the blood lysate procedure.

## Discussion

Haemoparasitic diseases have a considerable impact on the health and productivity of livestock and are responsible for remarkable economic losses^26^ with a very high rate of morbidity and mortality. These haemoparasitic infections frequently exhibit diverse and nonspecific clinical symptoms, making clinical diagnosis difficult. Moreover, common laboratory tests such as conventional parasitological techniques and serological techniques too have certain limitations.^2^^,^^4^^,^^5^ that can be overcome by molecular diagnostics like PCR assay but the biggest issues are its cost and labor-intensive DNA extraction process. In the present investigation, an attempt has been made to overcome such limitations in diagnosing haemoparasitic diseases in animals. Direct PCR, sometimes referred to as colony PCR, has long been used in microbiology and biotechnology laboratories to amplify the bacterial gene from a bacterial colony. Here, a small number of bacteria directly or bacterial lysate in boiling water are used as DNA template.^27^^,^^28^ However, these methods may not be universally applicable, particularly in cases involving parasites, encapsulated bacteria, or host tissues.In such instances, specific treatments of the samples are necessary to release the nuclear material from the cells. However, these treatments must not adversely affect the PCR results. In such cases, either alkaline solution (NaOH) was just utilized to treat the samples to amplify the targeted genes of *Campylobacter* from culture and *Babesia, Theileria, Hepatozoon*, and *Borrelia miyamotoi* in hard ticks^29–31^ or sodium dodecyl benzenesulfonate solution to extract the DNA for detection of bovine leukemia virus in bovine blood.^32^ Given that the majority of haemoparasites reside intracellularly, the initial step involves releasing them from the host cells. Additionally, blood contains various intrinsic factors such as hemoglobin, proteins, polysaccharides, lipids, and their conjugates, as well as extrinsic factors like EDTA or heparin, which can act as PCR inhibitors. These factors must be addressed to ensure the success of PCR analysis.^6^^,^^7^ NaOH is known to have good bleaching activity to lyse the cell membrane and as an alkaline reagent to increase the pH of the solution. PCR at alkaline pH is considered as advantageous. Bu et al.^7^ reported that at higher pH (9.1–9.6), the inhibitory action of PCR inhibitors present in blood can be overcome without affecting the amplification process of DNA. Many efforts have been made to directly amplify the targeted genes of the host by the use of blood samples as a starting material in PCR. For that, either PCR reagents were modified^17–20^ or minor processing of blood was done before use in PCR.^6^^,^^7^^,^^9–12^ Similarly, we also achieved the amplification of the host-associated gene (GAPDH) in a standard PCR process using blood lysate as template DNA that was either produced in nuclease-free water or with various graded concentrations of NaOH (5–80 mM). Direct blood did not show any amplification. The highest level of amplification was observed in blood lysate prepared from 55 mM NaOH. In contrast, no amplification of the piroplasm gene (18S rRNA) was observed in blood lysate prepared with water alone. However, a nearly identical level of amplification was detected in blood lysate prepared with NaOH concentrations ranging from 10 mM to 25 mM. Subsequently, decreasing trends in amplification were noted with higher NaOH concentrations. No amplification was found after 55 mM NaOH. This indicates that special treatments are required to release the parasite DNA from the cells where even a small quantity of NaOH is enough at a particular temperature. However, at higher pH, modification in PCR reagents such as a change in MgCl_2_ and primers concentration and the addition of PCR enhancer will help achieve the optimum amplification.^7^ In the present study also, we obtained optimum amplification of targeted genes in a PCR reaction mixture containing 2 mM of MgCl_2_, 20 pmole each of forward and reverse primers, and 0.4 M Betaine as PCR enhancer. The optimal amplification of targeted DNA fragments was achieved with 4 µl of blood lysate in a 25 µl PCR reaction, as opposed to using a higher volume of blood lysate. This phenomenon may be attributed to the adverse effects of increased alkalinity and the presence of additional blood-associated PCR inhibitors on the PCR assay.^7^

The minimum amount of blood required for host DNA amplification is sufficient to amplify the desired nucleotide sequence, but for haemoparasites, particularly those that are intracellular and typically have low parasitemia, a larger volume of blood may be necessary for blood lysate preparation to attain the desired result. In light of this, we observed that blood lysate prepared from 20 µl of blood in 200 µl of 15 mM NaOH gave the best amplification as compared to other combinations. Sensitivity and specificity are the two main facets of any diagnostic test. The sensitivity and specificity of the PCR assay mainly depend upon the primers, quality of DNA as template, and other PCR reagents at optimized PCR conditions. In the present study, we utilized in-house standardized PCR primers designed specifically to amplify the targeted DNA fragments of piroplasm (*Babesia/Theileria*), *B. bigemina, T. annulata, T. orientalis, T. evansi, Rickettsia/Anaplasma and E. canis.*^4^ On the other hand, the current approach can detect parasitaemia at incredibly low levels and is nearly as sensitive as the purified DNA-based assay. The same has been validated on field samples. As expected, all microscopically positive samples reacted positively in PCR too. So, the primary evaluation of the test was performed on microscopically negative samples. In microscopically negative blood samples, PCR based on purified whole blood genomic DNA and blood lysate was found to be equally effective for the detection of *T. annulata, B. bigemina, T. evansi, Rickettsials/Anaplasma, and E. canis*; however, the detection of piroplasm and *T. orientalis* was found to be somewhat less efficient but statistically non-significant. Further, the specific reactivity of the PCR in blood lysate was confirmed through random sequencing of the amplified product. It indicates the wider field applicability of the blood lysate technique for the diagnosis of haemoparasitic infections in animals.

Another goal of the current investigation was to lower the cost of diagnosis, and it was found that the blood lysate approach was less expensive in terms of sample preparation than commercial DNA isolation kit methods by more than 95%. It can also be used in laboratories with limited resources because it only needs basic equipment like a simple water bath, a simple mini centrifuge, and a vortexer. In contrast, highly sophisticated equipments are needed to purify DNA, including a high-speed centrifuge, a precise water bath, and a deep freezer (−20 °C). However, the cost of a PCR reaction for a sample was about 15% more than PCR on purified DNA due to additional expenditures in PCR reagents in the blood lysate procedure. The blood lysate method is still advantageous since it demands less pipetting and centrifugation, lowering cross-contamination risk. It is also simpler to use, poses minimal health hazards, and requires merely 10–15 min to process a blood sample for PCR.

## Conclusion

In conclusion, the currently developed and optimized blood lysate method is a viable alternative to purified whole blood genomic DNA for molecular detection (PCR) of haemoparasites such as *Babesia, Theileria, Trypanosoma* and Rickettsiales in cattle, buffaloes, horses, and dogs. It is fast, inexpensive, more convenient, and can be performed in laboratories with limited resources.

## Supplementary Material

Supplementary Table. 1..docx

## Data Availability

The data used to support the findings of this study are included within the article.
