# Supplementary material for: Molecular identification of haemoparasites in animals using blood lysate PCR: a quick and inexpensive alternative to purified whole genomic DNA
Source: Anim Biotechnol. 2024 Aug 13;35(1):2390935. doi: 10.1080/10495398.2024.2390935 (PMC12674356; doi:10.1080/10495398.2024.2390935)
Supplement: Supplementary Table. 1..docx [file LABT_A_2390935_SM0111.docx]

**Supplementary Table. 1**. Generation of nucleotide sequences after amplification of targeted DNA fragments in blood lysate PCR

| Haemoparasites | Host | GenBank Accession No. (NCBI, USA) | Nucleotide sequences generated |
| --- | --- | --- | --- |
| *Theileria annulata* | Cattle | [OQ991315](https://www.ncbi.nlm.nih.gov/nuccore/OQ991315) | 18S rRNA |
| *Theileria orientalis* | Buffaloes | [OQ992767](https://www.ncbi.nlm.nih.gov/nuccore/OQ992767) | 18S rRNA |
| *Theileria equi* | Horse | [OQ992771](https://www.ncbi.nlm.nih.gov/nuccore/OQ992771) | 18S rRNA |
| *Babesia vogeli* | Dogs | [OQ993069](https://www.ncbi.nlm.nih.gov/nuccore/OQ993069) | 18S rRNA |
| *Anaplasma* spp. | Cattle | [OQ993164](https://www.ncbi.nlm.nih.gov/nuccore/OQ993164) | 18S rRNA |
| *Anaplasma* spp. | Buffalo | [OQ996267](https://www.ncbi.nlm.nih.gov/nuccore/OQ996267) | 18S rRNA |
| *Ehrlichia canis* | Dogs | [OQ993340](https://www.ncbi.nlm.nih.gov/nuccore/OQ993340) | 16S rRNA |
| *Theileria annulata* | Cattle | OR039343 | cytochrome b (cytb) |
| *Theileria annulata* | Buffaloes | OR039341 | cytochrome b (cytb) |
| *Theileria orientalis* | Cattle | OR039344 | major piroplasma surface protein (MPSP) |
| *Theileria orientalis* | Buffaloes | OR039345 | major piroplasma surface protein (MPSP) |
| *Trypanosoma evansi* | Cattle | OR039342 | RoTat1.2 variable surface glycoprotein (VSG) |
| *Babesia bigemina* | Buffaloes | OR039350 | rhoptry-associated protein 1c |
